# Barriers to and facilitation of long-term physical exercise participation among Chinese adolescents with autism spectrum disorder: a parental perspective

**DOI:** 10.3389/fpsyg.2026.1797823

**Published:** 2026-05-08

**Authors:** Lei Xu, FengLing Zhang, XinWei Li

**Affiliations:** 1 School of Physical Education, South China University of Technology, Guangzhou, China; 2 Hubei Polytechnic Institute, Xiaogan, China; 3 Hunan Institute of Engineering, Xiangtan, China

**Keywords:** adolescents with ASD, barriers, facilitation, long-term physical exercise, parental perspective

## Abstract

From a parental perspective, this study explores the main barriers and facilitation strategies associated with long-term participation in physical exercise among Chinese adolescents with autism spectrum disorder (ASD). A qualitative research design was adopted, involving semi-structured interviews with parents of 19 adolescents with ASD who had participated in physical exercise for more than 3 years. Interview data were analyzed using reflexive thematic analysis. The findings indicate that adolescents with ASD encounter five major categories of barriers during sustained physical exercise participation: physical, cognitive, social communication, rule-related, and psychological adaptation barriers. In response to these challenges, five facilitation strategies were identified, including: improving rule design, adjusting instructional content and task difficulty, strengthening post-session tasks, refining training methods, and optimizing instructional delivery. Together, these findings provide practical insights for enhancing the effectiveness and sustainability of long-term physical exercise participation among adolescents with ASD.

## Introduction

Autism spectrum disorder (ASD) is a lifelong neurodevelopmental condition characterized by persistent deficits in social communication and interaction, alongside restricted and repetitive patterns of behavior (Francis et al., 2021). In recent decades, the global prevalence of ASD has increased steadily, making it an increasingly prominent public health concern (Zhu et al., 2025). Adolescence represents a critical developmental period during which behavioral patterns, lifestyle habits, and adaptive capacities gradually consolidate, with long-term implications for independence, social participation, and quality of life in adulthood. In China, the population of individuals diagnosed with ASD is both large and continuously growing (Jiang et al., 2024; Zhou et al., 2020), highlighting an urgent need for sustainable and developmentally appropriate support strategies tailored to Chinese adolescents with ASD.

Physical exercise has been widely recognized as a promising intervention for individuals with ASD (Jia et al., 2024; Rosenthal-Malek and Mitchell, 1997). However, despite growing evidence of its benefits, sustained participation in physical exercise remains a significant challenge in real-world contexts. Existing studies report low participation rates, limited exercise duration, and poor adherence among adolescents with ASD (Salar et al., 2024), suggesting that the effectiveness of physical exercise depends not only on its potential benefits but also on the conditions that support long-term engagement.

Previous research has identified a range of factors influencing physical exercise participation among individuals with ASD, including family and social support, school and community resources, and environmental conditions, as well as individual-level characteristics such as peer interaction difficulties and psychological stress (Gürkan and Kocak, 2023; Ince et al., 2025; Okkenhaug et al., 2024; Waddington et al., 2025). However, several important conceptual and empirical gaps remain. Existing studies have predominantly emphasized external contextual factors, with comparatively limited attention to internal and process-oriented challenges that emerge during sustained participation. Moreover, empirical evidence on how exercise practices are adapted in response to these challenges in everyday contexts remains scarce. In addition, research in non-Western settings–particularly China–is still limited, despite the potential influence of cultural, educational, and family environments on exercise participation among adolescents with ASD.

In response to these gaps, this study makes several contributions. First, it adopts a process-oriented perspective to examine how participation barriers emerge and interact over time, moving beyond static categorizations of influencing factors. Second, by drawing on parental accounts, the study provides a contextually grounded understanding of long-term participation from a family perspective. Third, it identifies and maps relationships between different categories of barriers and corresponding facilitation strategies, offering an integrative framework that links challenges with potential responses. Finally, by focusing on the Chinese context, the study contributes to expanding the evidence base beyond predominantly Western settings.

Guided by these aims, the present study employs a qualitative approach to explore barriers to long-term physical exercise participation among Chinese adolescents with ASD from a parental perspective and to identify potential pathways for supporting sustained engagement. Specifically, the study addresses the following research questions:

(1) What are the main barriers faced by adolescents with ASD during long-term participation in physical exercise?
(2) Based on parental observations and reflections, in what ways do current exercise practices for adolescents with ASD need improvement to encourage ongoing participation?

## Materials and methods

### Participants

Participants were parents affiliated with an autism association in Guangzhou, China, which provides services for children and adolescents with ASD. A purposeful sampling strategy was used to ensure that participants could provide rich and relevant insights into long-term exercise experiences of adolescents with ASD. Inclusion criteria required that: (1) parents were willing to participate in interviews and data verification; (2) the adolescent had engaged in regular physical exercise for at least 3 years (≥2 sessions per week, ≥1 h per session); (3) parents were highly familiar with and had supported more than half of their child’s exercise activities over the past 3 years; and (4) adolescents were aged 10–19 years.

The association initially recommended 25 eligible parents. After screening, 6 were excluded due to low participation willingness (*n* = 2), insufficient exercise duration (*n* = 1), limited familiarity with exercise participation (*n* = 2), or age ineligibility (*n* = 1). The final sample comprised 19 parents who met all inclusion criteria. This sampling approach does not aim for statistical representativeness but follows widely accepted qualitative principles, prioritizing depth, relevance, and richness of data over generalizability. In line with the concept of “information power” (Malterud et al., 2016), the sample size was considered adequate given the specificity of the sample, the relevance of participants’ experiences to the research aim, and the richness of the data collected. Recurring patterns across interviews further supported the adequacy of the sample for the study’s analytic aims.

Among the 19 parents interviewed, 5 were fathers, and 14 were mothers (Table 1). All participant identifiers are presented using the initials of the adolescents’ names, with the final letter replaced by an asterisk (*) to ensure anonymity and confidentiality. The adolescents with ASD included 17 males and 2 females, with a mean age of 16.05 years (range: 12–19 years). Information regarding ASD severity was derived from parental reports of adolescents’ clinical diagnoses and functional characteristics, with reference to the DSM-5 descriptions of autism spectrum disorder support levels (American Psychiatric Association, 2013). 13 adolescents were described as presenting moderate functional difficulties, while 6 adolescents exhibited mild to moderate functional difficulties. Support needs ranged from Level 1–2 (some support) to Level 2 (substantial support). Language and social impairments were most common, followed by intellectual and emotional or adaptive difficulties (language: *n* = 9; social: *n* = 5; intellectual: *n* = 2; emotional/adaptive: *n* = 3). All adolescents had participated in regular physical exercise for 3–8 years (mean = 5.4 years), engaging 2–7 times per week for 1–3 h per session. The physical exercise activities most frequently participated in by adolescents with ASD in this study included soccer, basketball, running, badminton, swimming, and roller skating. This study was approved by the Ethics Committee for Scientific Research of Guangzhou College of Commerce (No. GCC2023-003).

**TABLE 1:** Basic information and physical exercise of adolescents with ASD.

Interviewee	Relationship	Age	ASD severity	Key difficulties	DSM-5 level	Years of exercise	Frequency/ week	Duration/session	Exercise types
ZR*	Father–son	12	Moderate	Emotional disorder	2	3	2–7	1.5–2 h	Roller skating, soccer, basketball, running
CJ*	Mother–daughter	19	Moderate	Intellectual disability	2	7	3	1.5–3 h	Swimming, badminton, running
HZ*	Father–son	17	Moderate	Language disorder	2	7	3–7	2–3 h	Swimming, badminton
LX*	Mother–son	12	Mild–moderate	Social communication disorder	1–2	4	3	1–2.5 h	Soccer, basketball
ZX*	Father–son	19	Moderate	Language disorder	2	4	2	1–2 h	Soccer
NZ*	Mother–daughter	18	Moderate	Intellectual disability	2	8	2–7	1–2 h	Running, basketball
HY*	Mother–son	19	Moderate	Language disorder	2	8	4–7	1.5–2 h	Table tennis, badminton
H*	Mother–son	19	Mild–moderate	Social communication disorder	1–2	6	2–7	1–2 h	Soccer, running
ZY*	Mother–son	19	Moderate	Language disorder	2	8	7	1 h	Soccer, running
ZE*	Mother–son	18	Moderate	Delayed response	2	3	2–7	1.5–2 h	Basketball, soccer
LZ*	Mother–son	19	Mild–moderate	Social communication disorder	1–2	5	2–3	1–1.5 h	Basketball, soccer
DW*	Mother–son	17	Moderate	Language disorder	2	8	2–3	2–3.5 h	Running
LB*	Mother–son	12	Moderate	Emotional disorder	2	4	3	1–1.5 h	Soccer, basketball
JQ*	Mother–son	19	Mild–moderate	Social communication disorder	1–2	5	2–7	1–2 h	Basketball, running
FY*	Mother–son	19	Moderate	Language disorder	2	4	5	2–2.5 h	Basketball, swimming
WH*	Father–son	17	Moderate	Language disorder	2	7	3–4	1–1.5 h	Running, soccer
C*	Mother–son	19	Mild–moderate	Social communication disorder	1–2	4	2–3	1–1.5 h	Running, cycling
HJ*	Father–son	12	Mild–moderate	Social communication disorder	1–2	4	3	1–1.5 h	Running, basketball
YM*	Mother–son	18	Moderate	Language disorder	2	8	3	1–2 h	Running

ASD severity was derived from parental reports of adolescents’ clinical diagnoses and functional characteristics.

*Denotes that the final letter of each participant’s name has been replaced for anonymization purposes.

### Procedure

To ensure rigorous and ethical data collection, all interviews were conducted face-to-face in offline settings under appropriate safety precautions. A total of 19 interviews were conducted between December 11, 2023, and January 20, 2024, each lasting approximately 35–60 min. Interview locations were arranged based on participant preference and included football fields (*n* = 4), cafés (*n* = 3), metro stations (*n* = 3), KFC restaurants (*n* = 3), parents’ or adolescents’ workplaces (*n* = 4), the researcher’s university (*n* = 1), and Dashihou Restaurant (*n* = 1).

Interviews were conducted using a semi-structured protocol and were audio-recorded in full. Field notes were taken during and immediately after each interview to capture nonverbal cues, contextual details, and initial impressions. At the beginning of each interview, parents provided background information about their adolescents, including primary symptoms, behavioral characteristics, and patterns of physical exercise participation (e.g., frequency, duration, and activity types). The interviews then focused on two core areas: perceived barriers to long-term participation in physical exercise and potential strategies for optimizing current exercise practices. Example questions included: “What has been the greatest barrier or difficulty your child faces in participating in physical exercise?” and “In what ways can physical exercise be optimized to better support your child’s daily behaviors?” Follow-up and probing questions were used to clarify responses and ensure depth and richness of the data.

All interviews were conducted by trained graduate students with backgrounds in physical education for adolescents with ASD, who had prior experience collaborating with the local autism association. This prior engagement facilitated rapport-building and contextual understanding but may also have introduced potential bias. To address this, reflexive practices were integrated throughout the research process. Interviewers remained attentive to their roles and sought to minimize leading questions or assumptions during data collection. Field notes included both contextual observations and reflective comments. During data analysis, analytic memos were used to document evolving interpretations, and regular team discussions were conducted to critically examine assumptions and enhance reflexive awareness. These strategies contributed to the credibility and trustworthiness of the findings.

### Transcription and translation

All interview recordings were transcribed verbatim in Chinese by members of the research team shortly after each interview session. To ensure transcription accuracy, the transcripts were cross-checked against the original audio recordings by a second researcher. When necessary, ambiguities in speech or contextual meaning were clarified by referring to the field notes taken during the interviews.

For the purposes of reporting in this manuscript, selected excerpts were translated into English. The translation was conducted by bilingual researchers familiar with both the study context and terminology related to autism research. To ensure that the original meanings were preserved, a subset of the translated excerpts was independently back-translated into Chinese. Any discrepancies were discussed and resolved through team discussions until consensus was reached.

### Data analysis

The interview data were analyzed using reflexive thematic analysis, following Braun and Clarke (2006, 2019). All interviews were transcribed verbatim and repeatedly read to ensure data familiarization.

Analysis was conducted in an inductive and data-driven manner. Coding was performed at the level of meaning units within each transcript. Initial codes were generated through close, line-by-line engagement with the data, focusing on segments of participants’ accounts relevant to barriers to long-term physical exercise participation and strategies for optimizing engagement. Codes were grounded in participants’ own language, and multiple codes were assigned to the same segment when different meanings were present. For example, statements describing “not understanding the rules of the activity” were initially coded as difficulty understanding rules, which was later organized under rule-related barriers.

Coding and theme development were iterative and reflexive processes. Codes were first examined and compared within and across transcripts, then grouped into preliminary categories based on shared meanings. These categories were subsequently reviewed and refined through repeated comparison with the original data, during which some codes were merged, separated, or redefined to better capture participants’ meanings. Through this iterative process, broader patterns of shared meaning were developed into candidate themes.

Themes were not directly derived from the data but were actively constructed through interpretive analysis. Candidate themes were reviewed against coded extracts and the full dataset to ensure coherence, internal consistency, and distinctiveness between themes. This review process resulted in the refinement, renaming, and final definition of themes.

Throughout the analysis, the research team engaged in regular discussions to critically reflect on interpretations and consider alternative readings of the data. These discussions aimed to enhance reflexive engagement rather than achieve inter-coder agreement, consistent with the epistemological foundations of reflexive thematic analysis.

Analytic memos were maintained throughout to document coding decisions, emerging interpretations, and theme development. Initial coding was conducted by the first author, with ongoing interpretive input from the research team. Final themes were defined and named to reflect an interpretive understanding of parental perspectives on barriers to and optimization of sustained physical exercise among adolescents with ASD.

### Trustworthiness

To enhance the credibility of the findings, several strategies consistent with reflexive thematic analysis were employed. First, coding and theme development were conducted through an iterative and reflexive process, with ongoing analytic discussions among members of the research team to deepen interpretation and challenge assumptions. These discussions were not intended to achieve consensus, but to support reflexive engagement with the data and enhance interpretive depth. Second, member reflections were conducted after the themes had been developed. Preliminary themes were shared with five parents (ZR*, LX*, H*, ZY*, JQ*), who were invited to reflect on whether the interpretations resonated with their experiences. Their feedback generally supported the relevance of the themes, and no substantial revisions were required.

## Results

### Barriers to long-term participation in physical exercise among adolescents with autism spectrum disorder

This study identified five major categories of barriers faced by adolescents with ASD during participation in physical exercise (Table 2): physical factors, cognitive factors, social communication factors, rule-related factors, and psychological adaptation factors. It is worth noting that the frequencies reported below indicate the number and proportion of parents mentioning each theme, rather than population prevalence.

**TABLE 2:** Themes and examples of barriers to long-term physical exercise participation among adolescents with autism spectrum disorder.

Theme	Specific manifestations	Parents mentioning the theme	Frequency (percentage)	Illustrative quotation
Physical factors	Motor coordination; movement imitation; fine motor skills;	WH*, LZ*, LB*, JQ*, HY*, HZ*, H*, FY*	8 (42%)	“The greatest difficulty, I think, is that there are some movements he simply cannot do. His limbs are relatively clumsy and uncoordinated… The movement itself is not very difficult. I see other children performing it quite smoothly, so poor coordination really limits his ability to exercise.” (JQ*)
Cognitive factors	Attention; information processing; organizational ability	WH*, LZ*, H*, HZ*, JQ*, LX*, NZ*, C*	8 (42%)	“If the waiting time is long, his attention will become distracted. For example, if there are only five children in a line taking turns to do an action, he can maintain attention. But if there are ten or fifteen, it is very easy for him to lose focus.” (HZ*)
Social communication factors	Social initiative; teamwork; verbal expression	DW*, H*, LX*, ZR*, ZY*, YM*	6 (32%)	“He just cannot find peers to interact with. As I mentioned, because of his social difficulties, he will not take the initiative to communicate with classmates. His language expression is also problematic–sometimes he cannot express what he wants to say clearly.” (LX*)
Rule-related factors	Difficulty understanding rules	CJ*, HY*, JQ*, ZY*, C*	5 (26%)	“Most children in this group have intellectual impairments, so when the rules are particularly complex, they simply cannot understand them.” (JQ*)
Psychological adaptation factors	Fearfulness; unfamiliar environments; anticipation of future situations	HJ*, C*, HY*, ZR*	4 (21%)	“Some children are more timid. The bolder ones will kick the ball, but the timid ones just run along without knowing what they are supposed to do or why they are running.” (HJ*)

*n* (%) indicates the number and proportion of parents mentioning the theme.

*Denotes that the final letter of each participant’s name has been replaced for anonymization purposes.

### Physical factors

Regarding physical barriers, eight parents (42%) reported that adolescents with ASD experienced varying degrees of physical difficulty during physical exercise participation. These difficulties were mainly reflected in three aspects. First, insufficient motor coordination. Parents perceived that their adolescents often displayed movements that appeared clumsy or rigid, which is consistent with previous research on motor characteristics in ASD (Gowen et al., 2023). One parent noted, “*Their coordination ability is relatively weak. Movements are uncoordinated, and fine motor skills are not well developed. This is probably related to congenital differences in neurodevelopment, and many movements seem to never reach a proficient level*” (WH*). Second, limited imitation ability. Parents described that the acquisition of physical skills–typically involving observation, understanding, and reproduction of demonstrated movements–was particularly challenging for their adolescents. Several parents perceived that their children had difficulty identifying key movement components or grasping the implicit structure of actions. As one parent explained: “*For some movements, he just can’t grasp the key point. No matter how he tries, it’s incorrect. Even when the teacher or classmates demonstrate, he still can’t imitate it. I don’t know whether it’s due to limited ability or distraction*” (LB*). Third, insufficient fine motor skills. Parents reported persistent difficulties in hand dexterity and fine motor coordination, even after prolonged exercise participation, which aligns with existing findings in the literature (Simarro Gonzalez et al., 2024). One parent stated: “*Even after exercising for a long time, improvements in fine motor movements are still limited, and I don’t know how to help improve them*” (LB*). Overall, both parental perceptions and prior research suggest that the acquisition and execution of physical skills rely heavily on motor coordination, fine motor control, and imitation ability (Zhao et al., 2021). Parents reported that limitations in these areas hinder accurate movement execution and the ability to adjust skills during physical exercise. These observations align with existing literature indicating that deficits in motor coordination and fine motor control can lead to inaccurate movements and poor rhythmic integration (Alsaedi, 2020), while weaker imitation abilities make it more difficult for individuals with ASD to effectively learn and refine physical skills through observing teachers or peers (McWeeny et al., 2025).

### Cognitive factors

With regard to cognitive barriers, eight parents (42%) reported that their children experienced notable difficulties in this domain, which were mainly manifested in three aspects. First, insufficient attentional capacity. Parents perceived that their adolescents were highly sensitive to external stimuli and were easily distracted by environmental factors such as sounds, lighting, and moving objects. One parent noted: “*He is easily distracted. When running outdoors, cars and advertisements nearby are very attractive to him*” (H*). Other parents reported that when the environment was overly noisy, “*the child’s attention is easily disrupted; since it is already difficult to sustain attention, once it is interrupted, it becomes even harder to refocus*” (JQ*). In addition, parents described limited attentional endurance in some adolescents, which they perceived as making it difficult for their children to maintain focus for extended periods even in relatively low-distraction environments. As one parent described: “*His sustained attention is weak. It’s hard for him to focus on a single movement for a long time–after doing it briefly, he becomes tired or mentally disengaged*” (WH*). Second, reduced information processing capacity. Parents commonly reported that although their children appeared to understand what was being asked of them, they had difficulty rapidly translating verbal instructions into effective motor responses. One parent stated: “*Sometimes he can’t immediately understand and process the information–it’s like a short circuit*” (LZ*), while another noted, “*There are some techniques he really can’t master, because he doesn’t necessarily understand them*” (NZ*). Third, deficits in organizational ability. According to parents’ descriptions, some adolescents with ASD had difficulty completing tasks in a step-by-step manner and lacked coherent planning of action sequences. One parent explained: “*He doesn’t know what to do first and what to do second. There’s a lack of logical structure in how he approaches tasks*” (LX*). Overall, parents perceived that challenges related to attention, information processing, and organization interfered with their adolescents’ engagement in physical exercise. From an interpretive perspective, the execution of physical skills requires the active involvement of cognitive processes, including sustained attention, information processing, and the organization and sequencing of actions (Chen et al., 2023; Qi et al., 2025). During motor skill performance, individuals must process external stimuli, translate instructions into appropriate motor responses, and maintain focus throughout the task. However, adolescents with ASD often struggle to sustain attention in environments with multiple stimuli, have difficulty converting external instructions into coordinated movements, and experience challenges in planning and organizing movement sequences. It is important to note that these explanatory links represent theoretical interpretations that extend beyond parents’ direct observations and should be understood as contextualized insights rather than definitive causal conclusions.

### Social communication factors

With respect to social communication barriers, six parents (32%) identified difficulties in this domain. These challenges were primarily reflected in three aspects. First, limited social initiative. Parents perceived that some adolescents were reluctant to actively approach peers, initiate interactions, or engage in group activities during physical exercise. One parent noted, “*He does not take the initiative to communicate with classmates*” (LX*). Another parent reported that, compared with typically developing peers, the child still struggled to initiate greetings or join conversations naturally. Although progress had been observed, “*there is still a long way to go before reaching age-appropriate social competence*” (ZR*). Second, parents described team-based sports as particularly challenging for their children, as these activities require coordination with others and responsiveness to group dynamics. As one parent stated, “*Team coordination is a very big obstacle for them*” (H*). Third, limited expressive language ability. Some parents reported that their children often “*cannot express themselves clearly*” and “*have difficulty using appropriate and precise language to convey needs or emotions*” (LX*). Parents perceived that expressive difficulties made it harder for their adolescents to clearly communicate intentions during physical activities, which in some cases affected task completion. This limitation may hinder adolescents with ASD from effectively expressing themselves in movement contexts, thereby influencing how tasks are carried out. Overall, parents suggested that social communication challenges shaped their adolescents’ participation in physical exercise, particularly in interactive or cooperative situations. From an interpretive perspective, physical exercise is inherently social in nature (Macintyre, 2000), requiring communication and coordination with others in both individual and team-based activities. Difficulties in expressive language and social understanding may contribute to slower responses or more passive participation during cooperative tasks. Notably, this represents only a tentative inference derived from parental perceptions, rather than definitive evidence establishing a direct causal relationship.

### Rule-related factors

Concerning rule-related barriers, five parents (26%) reported that adolescents with ASD experienced difficulties when engaging in sports with complex structures. Based on parents’ observations, these difficulties were not limited to understanding the verbal expression of rules, but were also associated with challenges in integrating information during dynamic sports contexts that involve multiple rules. Several parents described these difficulties, noting that “*she cannot understand rules that are too complex*” (CJ*), “*when the rules are particularly complicated, they simply cannot grasp them*” (JQ*), and “*once the rules become complex, problems with understanding arise*” (ZY*). Parents also suggested that sports with relatively simple rules and limited interaction, such as one-on-one activities like badminton, seemed easier for their children. In contrast, team sports such as soccer, which involve multiple participants and rapidly changing situations, were perceived as more demanding. As one parent explained: “*Soccer has more rules, so it’s harder for him to understand; badminton has simpler rules, which makes it easier for him*” (HY*). From parents’ perspectives, understanding and adhering to rules appeared to influence adolescents’ participation in physical activities. As a defining feature that distinguishes sports from other forms of activity, rules require individuals to comprehend and follow them in order to participate effectively. Parents observed that their adolescents sometimes experienced difficulties in this regard, which could affect their engagement during physical exercise. However, interpretations that attribute these difficulties to limitations in contextual understanding or cognitive flexibility–such as explaining rule violations or rigid behavioral responses–remain speculative (Uddin, 2021), as such underlying mechanisms cannot be directly established from parental reports alone and should therefore be viewed as contextualized explanations rather than definitive causal conclusions.

### Psychological adaptation factors

Concerning psychological adaptation–related barriers, four parents (21%) reported that adolescents with ASD experienced difficulties in emotional security, environmental adaptation, and anticipation of future events. First, when confronted with physically demanding tasks, parents observed that their children often showed fearfulness or avoidance behaviors. For example, “*in competitive situations or activities that require independent decision-making, they may withdraw proactively, hesitate to perform key movements, and struggle to respond appropriately to situational changes*” (HJ*). Second, parents perceived that adolescents with ASD had limited adaptability to unfamiliar exercise environments. Parents reported that their children “*tended to feel more relaxed in familiar settings or fixed teams; however, once placed in novel contexts, their difficulties with environmental adaptation rapidly intensified feelings of discomfort and anxiety*” (HY*). Third, parents reported challenges in anticipating future events, describing that adolescents with ASD “*worrying about and feeling fearful toward unknown situations*” (ZR*), and they often required advance notification or preparation to reduce anxiety. From parents’ perspectives, these observations suggest that adolescents with ASD may experience challenges related to psychological readiness and flexible coping during physical exercise, particularly when faced with uncertainty. The dynamic and unpredictable nature of physical exercise contexts requires individuals to respond flexibly to changing situations and adjust their self-perceptions and behavioral strategies, making psychological adaptation an important factor in sustained participation (Hu et al., 2025). However, for adolescents with ASD, limited psychological readiness may make it difficult to effectively cope with such demands, potentially affecting their ability to gain positive participation experiences and maintain ongoing engagement. It is worth noting that interpretations regarding how these factors influence sustained participation or overall engagement remain speculative, as these explanatory links extend beyond parents’ direct observations and should be understood as contextualized rather than causal conclusions.

### Facilitation pathways for long-term participation in physical exercise among adolescents with autism spectrum disorder

Five major facilitation pathways supporting sustained physical exercise participation among adolescents with ASD were identified (Table 3). These pathways were: improved rule design, adjustment of instructional content and task difficulty, structured post-session tasks, refinement of training methods, and optimization of instructional delivery.

**TABLE 3:** Themes and examples of facilitation pathways for long-term physical exercise participation among adolescents with autism spectrum disorder.

Theme	Specific manifestations	Parents mentioning the theme	Frequency (percentage)	Illustrative quotation
Rule design	Rule simplification; staged presentation of rules; Multimodal rule representation	HY*, JQ*, ZR*, ZY*, ZX*, C*, HJ*	7 (37%)	“If the rules can be simplified and the instructions made simpler, it would be much better. When the rules are simple, they can better understand what is being taught.” (JQ*)
Instructional content and task difficulty	Increased balance-related activities; reduced task difficulty; inclusion of competitive elements	CJ*, DW*, LZ*, LB*, NZ*	5 (26%)	“If the tasks are too complex, she cannot understand them at all, which makes them meaningless. I hope the exercises can be simpler, such as running or basketball, with fewer technical requirements and within her ability range.” (CJ*)
Post-session tasks	Clear operational guidance; structured post-session practice	FY*, H*, LB*, LZ*, WH*	5 (26%)	“If tasks are assigned, and if conditions allow, we are willing to support them in completing the practice.” (FY*)
Training methods	Increased feedback; movement decomposition; repetitive practice	FY*, H*, DW*	3 (16%)	“It is best to divide movements into very small steps, because their comprehension is limited. Without breaking them down, they can only imitate superficially and the effect is not achieved.” (FY*)
Instructional delivery	Slower pace of instruction; repeated instructions; concise language	DW*, HY*, LZ*	3 (16%)	“Instructions must be simple, clear, and explicit. Experienced coaches give concise instructions, progress step by step in each session, and have clear teaching objectives.” (HY*)

*n* (%) indicates the number and proportion of parents mentioning the theme.

*Denotes that the final letter of each participant’s name has been replaced for anonymization purposes.

### Improving rule design

Seven parents (37%) referred to rule design as a key theme during the interviews. Their suggestions for optimization mainly focused on three aspects: rule simplification, staged presentation of rules, and multimodal rule representation. First, rule simplification was emphasized by most parents. Condensing rules into core elements and reducing linguistic complexity were perceived as helpful for adolescents with ASD to better grasp the structure of physical exercise tasks. As one parent noted, “*Rules should be simplified… if a single word can be used, there is no need to say too much*” (JQ*). Second, parents highlighted the importance of staged rule presentation, in which rules are introduced gradually. This approach starts with the most basic and easily understandable sub-rules and progressively incorporates additional requirements as the adolescent’s abilities improve. For example, one parent explained, “*At the beginning, it was enough for him to just hit the ball over the net; later, we introduced boundaries and serving lines… adding them step by step*” (HY*). Such a progressive presentation of rules may effectively prevent cognitive overload caused by the simultaneous input of multiple pieces of information (Van Merrienboer and Sweller, 2005). Third, multimodal rule representation was frequently mentioned. Many parents pointed out that adolescents with ASD rely heavily on visual cues and tend to process information through a form of “visual thinking” (Kunda and Goel, 2011). As one parent described, “*His thinking works like a series of pictures… he has learned with cards since childhood*” (HY*). Presenting rules through visual aids (e.g., whiteboard diagrams, field maps, directional arrows, color coding), demonstration-based practice, or gamified formats was perceived to significantly enhance comprehension. For instance, “*Concepts like offside are very difficult to explain verbally, but they can be integrated through games*” (ZR*). These three strategies converge on a central logic: by adjusting the content, structure, and mode of presentation, rules can be made more “comprehensible, decomposable, and perceptible,” thereby lowering the threshold for learning and enhancing the quality of participation. This not only reflects parents’ adaptive adjustments in practice but also suggests that, in designing physical activity programs for adolescents with ASD, rules are not merely behavioral constraints but also important instructional content that requires pedagogical consideration.

### Optimizing instructional content and task difficulty

Five parents (26%) provided suggestions related to instructional content and task difficulty, emphasizing the need to tailor physical exercise programs to the characteristics of adolescents with ASD. These optimization strategies primarily focused on three aspects: increasing balance training, reducing task difficulty, and introducing competitive elements. First, increasing balance training was frequently mentioned. Some parents noted that adolescents with ASD often exhibit weaknesses in vestibular processing, core strength, and overall motor coordination, which might lead to difficulties in postural control across various physical activities. For example, one parent stated, “*She still cannot ride a bicycle… her balance is very poor, and I hope more related training can be included in class*” (NZ*). Balance training has been reported to support motor control, spatial awareness, and motor planning, particularly among individuals with ASD (Stins and Emck, 2018). From an interpretive perspective, incorporating balance-related exercises (e.g., stability training, balance board activities, single-leg stance) could help improve fundamental motor skills and build movement confidence, which may potentially in turn increase willingness to engage in more complex physical exercise. Second, reducing task difficulty was considered essential. Parents generally believed that some physical activities are overly complex or require advanced technical skills, which can potentially result in comprehension difficulties, increased frustration, and even refusal to participate. As one parent explained, “*When it is too complex, she simply cannot understand it… I hope the activities can be simpler*” (CJ*). In instructional practice, simplifying movement structures and designing tasks within the adolescent’s zone of proximal development might help ensure that they can follow instructions, keep pace with the activity, and experience success at a manageable level of challenge. Finally, parents suggested introducing competitive elements once basic skills had been established. Incorporating small-scale competitions or group-based contests were perceived as a way to promote skill transfer, enhance motivation, and foster a sense of teamwork. For instance, one parent noted, “*Three-on-three or five-on-five basketball games can test what they have understood and help them develop a sense of competition*” (LB*). Competitive contexts may stimulate engagement, motivation, and task involvement among adolescents with ASD (Jachyra et al., 2021), and also allow parents to observe learning outcomes. “*Competitions can motivate them… and help parents see the results*” (LZ*). However, it is important that such activities be accompanied by clear rules and simplified scoring systems to ensure accessibility and reduce unnecessary pressure. These three types of recommendations complement one another in function: balance training focuses on building foundational motor abilities, reducing task difficulty emphasizes the alignment between task demands and individual capability, while the introduction of competitive elements primarily serves to stimulate and sustain participation motivation. Collectively, they point to a central goal–through layered design and gradual adjustments, physical activities can be structured to be both achievable and sufficiently challenging and engaging, thereby achieving a balance between skill development and participation experience.

### Enhancing post-session tasks

Five parents (26%) referred to post-session tasks as an important theme, indicating that adolescents with ASD require more systematic practice beyond formal training sessions. Parental feedback primarily focused on two aspects: providing clear operational guidance and increasing opportunities for post-session practice. First, parents emphasized the need for clear and practical guidance for post-session activities. As one parent noted, “*Many parents do not know how to help improve their child’s performance or what exactly they should ask the child to do at home*” (FY*). Accordingly, parents suggested that teachers or coaches provide concise and feasible practice instructions after each session, including movement steps, practice frequency, and key precautions. Such guidance may enable families to more effectively support skill consolidation in daily contexts. Second, parents highlighted the importance of assigning structured post-session practice tasks. As one parent explained, “*When families and schools work together… progress tends to be much faster*” (LB*). Structured post-session practice not only increases opportunities for repetition but also facilitates the transfer and consolidation of learned skills across different settings. In addition, many parents reported that adolescents with ASD tend to demonstrate higher compliance and willingness to complete tasks assigned by teachers or coaches than those suggested by parents. As one parent observed, “*When the teacher assigns homework, he feels more pressured and is more willing to complete it… but when it comes from parents, he is much less cooperative*” (LZ*). Based on this characteristic, teachers or coaches may leverage their authoritative role by assigning post-session tasks to enhance practice effectiveness among adolescents with ASD. The above recommendations form a relatively coherent support chain in terms of function: clear guidance addresses the question of “how to perform,” while structured tasks address “what to do and how much to do.” Together, these elements help build a continuous learning system extending from the classroom to the home, thereby promoting the consolidation and transfer of physical skills.

### Refining training methods

Three parents (16%) referred to training methods as a key theme, with their core needs focusing on increasing feedback, decomposing movements, and emphasizing repetitive practice. First, parents emphasized the importance of increased feedback. As one parent stated, “*I really hope there can be more feedback… such as my child’s initiative, compliance with instructions, and motor performance in this session. A simple checklist would be enough… so that we can understand how he has been doing recently*” (H*). Providing standardized feedback records to parents could enhance home–school collaboration and enable parents to offer more targeted support. Second, parents highlighted the need for movement decomposition. As one parent explained, “*Movements are not corrected all at once… it is better to break them down into small components and build them up from simple to complex*” (FY*). This reflects the difficulties adolescents with ASD often experience in movement imitation and the organization of sequential actions (Bar Yehuda and Bauminger-Zviely, 2024; Wadsworth et al., 2017). Decomposed instructional approaches might reduce task complexity while strengthening mastery of individual components, thereby improving overall skill acquisition. Finally, parents stressed the importance of repetitive practice. As one parent noted, “*Movements need to be practiced many times, over and over again, rather than being taught all at once*” (H*). Repetitive practice aligns with the preference of adolescents with ASD for high-frequency repetition, stable environments, and fixed routines during learning (Omori, 2025). These three training methods complement one another in function: feedback provides informational support that allows the training process to be monitored and adjusted; movement decomposition reduces learning difficulty, enabling complex skills to be acquired step by step; and repetitive practice reinforces learning outcomes, facilitating skill consolidation and internalization. Collectively, they form a progressive training cycle of “understanding–practice–consolidation,” which helps reduce learning barriers while enhancing the stability and transferability of acquired skills.

### Optimizing instructional delivery

Three parents (16%) referred to instructional optimization as an important theme, with suggestions primarily focusing on slowing down instruction, repeating instructions, and simplifying instructional language. These recommendations reflect typical characteristics of adolescents with ASD in terms of information processing, language comprehension, and executive functioning. First, parents emphasized the importance of slowing the pace of instruction. They noted that when instructions are delivered too quickly, adolescents with ASD often fail to complete auditory processing and semantic comprehension in time, making it difficult for them to keep up with the pace of the session. As one parent stated, “*When instructions are given too fast, they simply cannot follow and do not understand what is being said*” (DW*). Second, parents highlighted the value of repeating instructions. Repeated prompts and guided repetition were perceived as effective strategies for improving the accuracy of instruction comprehension. As one parent explained, “*Instructions need to be repeated during class… they should be told several times, and the child must repeat them back before they can gradually understand*” (DW*). This aligns closely with the executive functioning difficulties commonly observed among adolescents with ASD (Seng et al., 2021). Repetition and verbal rehearsal may help consolidate information and reduce task deviation. Third, parents stressed the need for concise and simplified instructional language. Simplified instructions can effectively reduce cognitive load and enhance task execution efficiency. Parents emphasized that teachers’ or coaches’ language should be as brief, direct, and low-load as possible. As one parent noted, “*Instructions should be short and concise… too much information cannot be processed at once… short phrases are much easier for them to understand*” (LZ*). These three strategies form a teaching regulation system centered on “input optimization”: slowing down the pace provides sufficient time for information processing, repeating instructions enhances the accessibility and stability of information, and simplifying language reduces the threshold for comprehension. Working together, these approaches help align instructional input with individuals’ information processing pace, thereby improving the accuracy of instruction comprehension and the continuity of task execution.

### Mapping between barrier categories and corresponding pathways

The preceding analyses identified key participation barriers and a set of optimization pathways for supporting adolescents with ASD in physical exercise. To further clarify the relationships between barrier categories and facilitation strategies, a mapping analysis was conducted to examine how different barriers are linked to multiple, and sometimes overlapping, optimization pathways. Table 4 presents the mapping between participation barriers and corresponding facilitation strategies, with representative quotations from parents provided as supporting evidence. The mapping highlights a many-to-many relationship, in which individual barriers may be addressed by multiple strategies, and single strategies may simultaneously respond to different types of barriers.

**TABLE 4:** Potential optimization strategies mapping between barrier categories and corresponding pathways.

Barrier category	Corresponding facilitation strategies	Representative quotations
Physical factors	Training methods; instructional content and task difficulty	“Movements are best broken into very small steps; without decomposition, they can only imitate superficially.” (FY*); “Her balance is very poor; I hope more balance training can be included in class.” (NZ*)
Cognitive factors	Instructional delivery; instructional content and task difficulty	“Instructions need to be repeated several times; the child must repeat them back to gradually understand.” (DW*); “If tasks are too complex, she cannot understand… simpler activities like running or basketball within her ability range.” (CJ*)
Social communication factors	Instructional content and task difficulty; post-session tasks	“Small basketball games with 3–5 players help them experience competition and understand rules.” (LB*); “After class, practice requirements help children improve; cooperation between families and school accelerates progress.” (H*)
Rule-related factors	Rule design; instructional content and task difficulty	“Now, gradually introduce football rules… first allow them just to hit the ball over the net, later add boundaries and service lines.” (HY*); “Simplify instructions to short, concise phrases–too many words are hard to process.” (LZ*)
Psychological adaptation factors	Post-session tasks; instructional delivery	“Instructions must be simple, clear, and explicit. Experienced coaches progress step by step and have clear objectives.” (LZ*); “The experienced coach gives clear instructions. Each class progresses step by step and the teaching objectives are clearly defined.” (HY*)

The correspondences presented in this table reflect conceptual alignment based on parental reports and should not be interpreted as evidence of empirically validated effectiveness.

*Denotes that the final letter of each participant’s name has been replaced for anonymization purposes.

**FIGURE 1:**
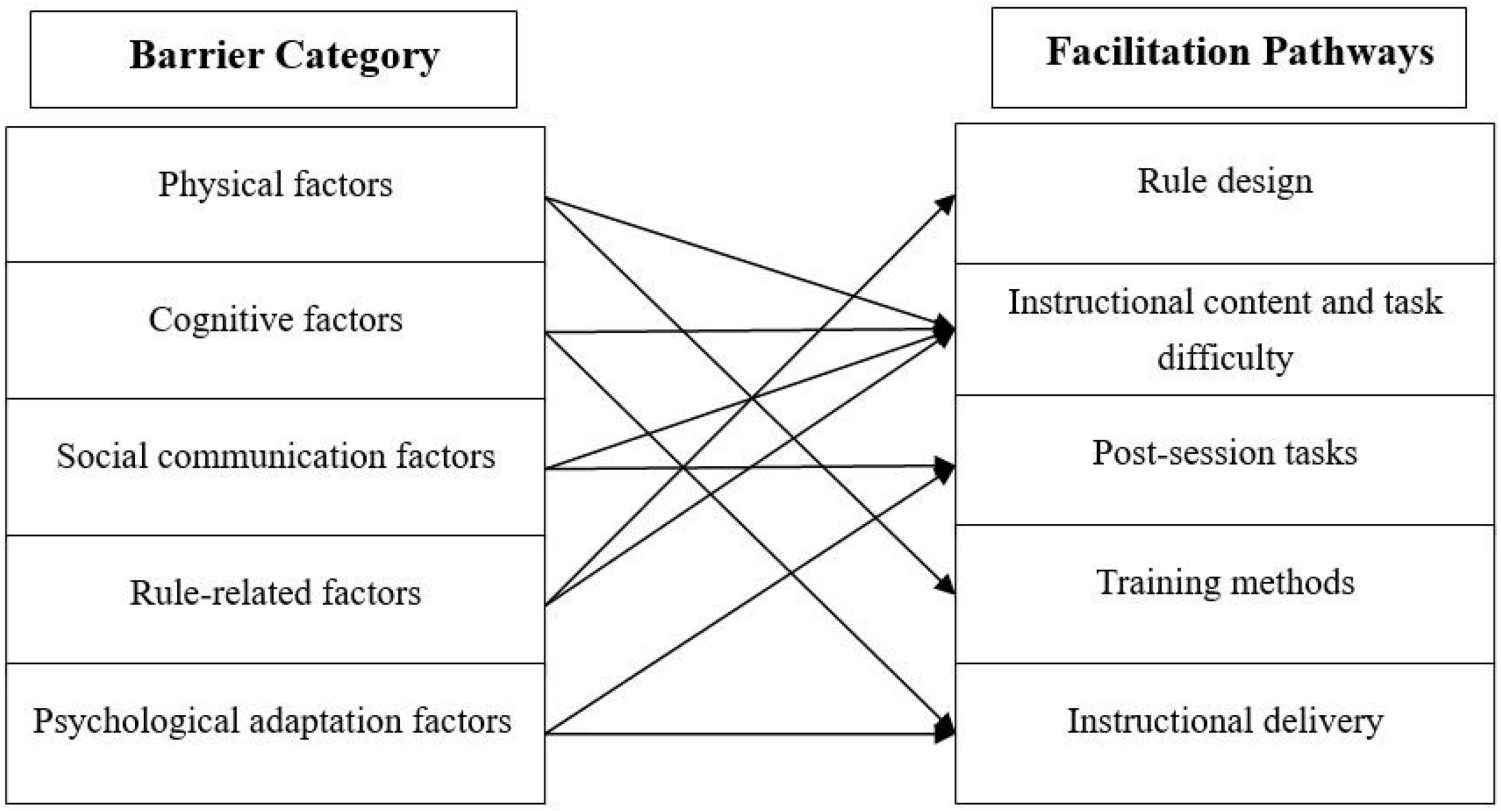
Conceptual mapping of barriers and facilitation strategies in physical exercise participation among adolescents with ASD.

Specifically, physical barriers were commonly associated with training methods and adjustments to instructional content and task difficulty. Strategies such as movement decomposition, repetitive practice, increased feedback, and the inclusion of balance-related activities were perceived as facilitating skill acquisition and improving movement performance. Cognitive barriers were primarily linked to challenges in understanding instructions and processing complex tasks (Ozonoff et al., 1991). These barriers were often addressed through modifications in instructional delivery, including a slower pace, repeated instructions, and concise language, as well as through simplifying instructional content and reducing task complexity. Social communication barriers were associated with both instructional content and post-session tasks. Parents emphasized the importance of incorporating cooperative or small-group competitive activities to promote peer interaction, alongside structured post-session practice with clear guidance to reinforce skills and facilitate social engagement. Rule-related barriers mainly stemmed from difficulties in understanding complex sports rules (Pan, 2010). Strategies related to rule design, such as rule simplification, staged presentation, and multimodal representation, were perceived as enhancing rule comprehension and supporting participation in structured activities. Psychological adaptation barriers were primarily linked to instructional delivery and post-session tasks. Clear and structured instruction, combined with guided post-session practice, was considered important for gradually building confidence and supporting adaptation to exercise environments. Overall, these findings suggest that facilitation strategies should not be understood as targeting isolated barriers, but rather as flexible and overlapping approaches that can address multiple domains of difficulty simultaneously, as illustrated in the conceptual model (Figure 1).

## Discussion

This qualitative study explored barriers to long-term participation in physical exercise among Chinese adolescents with ASD and identified potential pathways for optimizing exercise support. By grounding the analysis in parental experiences, the findings not only clarify key participation barriers but also highlight how specific instructional strategies may address these challenges. This integrated perspective provides a more comprehensive understanding of how sustained participation in physical exercise can be supported among adolescents with ASD.

### Barriers to long-term physical exercise participation among adolescents with ASD

This study categorized barriers into five interrelated domains: physical, cognitive, social communication, rule-related, and psychological adaptation factors. Prior studies have identified insufficient motor skills, executive function deficits, and social communication difficulties as key constraints on physical exercise participation among adolescents with ASD (Gürkan and Kocak, 2020; Must et al., 2015). The physical, cognitive, and social communication barriers identified in the present study align closely with these established findings, suggesting that such challenges remain relatively stable across contexts and developmental stages.

Building on this foundation, the study further extends existing research by identifying two additional categories–rule-related and psychological adaptation factors. Given that physical exercise often involves structured rules and task demands, difficulties in understanding and applying rules may represent a critical barrier, particularly in activities requiring higher levels of structure, such as team sports. At the same time, the identification of psychological adaptation factors highlights potential challenges related to fearfulness, unfamiliar environments, and anticipatory responses (Arnell et al., 2018). These challenges may be closely associated with perceived safety and prior participation experiences, both of which can influence sustained engagement.

Importantly, these barriers do not operate independently but interact in ways that shape participation experiences. For example, cognitive and rule-related difficulties may increase task complexity and uncertainty, which in turn can reduce confidence and contribute to maladaptive psychological responses. Similarly, limitations in motor competence may hinder successful participation experiences, thereby influencing willingness to engage and subsequent social interaction. These patterns suggest that barriers may form reinforcing cycles that constrain participation within a broader system of interacting factors.

Although this study did not adopt a longitudinal design, a process-oriented interpretation was used to contextualize participants’ accounts. Parents appeared to emphasize different barriers depending on participation experiences: earlier engagement was more frequently associated with challenges related to rule comprehension, cognitive load, and task complexity, whereas sustained participation was more often linked to psychological adaptation and participation experiences. However, these observations should be interpreted as retrospective sense-making rather than evidence of temporal change, as no systematic analysis of longitudinal processes was conducted. This perspective helps address a limitation in previous research, which has primarily focused on general barrier categories while paying less attention to how such barriers are experienced in the context of long-term participation (Okkenhaug et al., 2024).

### Analysis of optimization pathways for long-term physical exercise participation among adolescents with ASD

This study identified five optimization pathways for supporting sustained engagement in physical exercise among adolescents with ASD. These strategies are broadly consistent with established principles in special and inclusive education, as well as prior research, such as movement decomposition (Houston-Wilson and Lieberman, 2003) and the use of visual supports (Forbes and Yun, 2023). Strategies such as movement decomposition and task simplification align with the principle of scaffolding (Taber, 2018) by breaking down complex tasks into manageable components. Similarly, visual supports and multimodal instruction reflect structured teaching approaches (Preece and Almond, 2008) commonly applied in ASD interventions, while repeated practice and individualized pacing are consistent with principles of individualized support and skill generalization (Simpson et al., 2007; Wong et al., 2015).

Beyond this alignment, the findings highlight that these strategies serve different temporal functions within the participation process. Strategies such as rule simplification, movement decomposition, and concise instruction primarily facilitate initial engagement by reducing cognitive load and task complexity. In contrast, strategies including staged rule presentation, post-session tasks, and competitive formats rely on sustained participation and produce effects that emerge gradually over time. This distinction suggests that physical exercise participation should be understood as a progressively evolving process rather than a one-time intervention.

Importantly, the findings further point to a dynamic participation system in which barriers and strategies interact over time. Barriers co-occur across physical, cognitive, social communication, rule-related, and psychological adaptation domains, forming reinforcing cycles that constrain engagement. For instance, cognitive and rule-related difficulties may increase task uncertainty (Lange et al., 2018), which can reduce confidence and trigger maladaptive psychological responses, thereby further limiting participation. Within this system, strategies such as rule simplification, movement decomposition, and multimodal instruction can reduce cognitive load and enhance task clarity, interrupting these negative cycles and supporting initial engagement. As participation continues, changes occur not in the types of barriers themselves, but in their relative salience, with psychological adaptation and social communication challenges becoming more prominent. Correspondingly, strategies such as repeated practice, individualized pacing, structured feedback, and post-session tasks support skill acquisition and adaptive responses over time.

In addition, these strategies may exert cross-domain effects. For example, improving motor competence through structured practice may not only address physical barriers but also enhance confidence and reduce psychological adaptation challenges. Similarly, enhancing rule comprehension may facilitate smoother social interaction, thereby alleviating both rule-related and social communication barriers. These findings suggest that optimization strategies function as dynamic regulatory mechanisms that reshape interactions among barriers rather than addressing them in isolation.

Finally, the effectiveness of these strategies is shaped by contextual conditions. While some strategies (e.g., rule simplification and multimodal instruction) may be more readily implemented in resource-constrained or group settings, others (e.g., individualized feedback and post-session reinforcement) may require higher levels of instructional and family support. This highlights the need to adapt strategies flexibly across different contexts, taking into account variations in resources, instructional environments, and levels of support.

### Limitations

Although this study provides meaningful insights into barriers and facilitation pathways supporting long-term physical exercise participation among adolescents with ASD, several limitations should be acknowledged. First, the sample was drawn from a single autism association and consisted of parents of adolescents already engaged in sustained physical exercise, which may limit the diversity and representativeness of the findings. As such, the results are context-bound and should be interpreted within the specific institutional and participant context of this study. Future research should include larger and more diverse samples to enhance generalizability. Second, the study relied solely on parental reports, which may not fully capture the perspectives of other key stakeholders, such as teachers, coaches, and adolescents with ASD themselves. Future studies could adopt a multi-informant design to provide a more comprehensive understanding. Third, the study primarily reflects experiences related to aerobic and skill-based activities conducted in structured settings (e.g., school-based programs or organized training). Barriers to participation may differ across exercise types and contexts. For example, in structured group settings, social communication, rule-related, and cognitive barriers may be more prominent, whereas in less structured or home-based contexts, physical and psychological adaptation factors may play a greater role. Future research should further examine how participation barriers vary across different exercise forms and settings.

## Conclusion

This study examined the barriers to long-term physical exercise participation among adolescents with ASD and identified corresponding facilitation pathways from a parental perspective. The findings revealed that sustained participation is constrained by five interrelated factors: physical, cognitive, social communication, rule comprehension, and psychological adaptation. In response to these barriers, five practical facilitation strategies were proposed, including improved rule design, adjustment of instructional content and task difficulty, enhanced post-session tasks, refined training methods, and optimized instructional delivery. These findings provide practical guidance for supporting sustained physical exercise participation among adolescents with ASD and contribute to a deeper understanding of long-term exercise engagement in real-life contexts.

## Data Availability

The raw data supporting the conclusions of this article will be made available by the authors, without undue reservation.
